# Sliding mode control with an adaptive switching power reaching law

**DOI:** 10.1038/s41598-023-43304-6

**Published:** 2023-09-27

**Authors:** Shijiao Wang, Chengming Jiang, Qunzhang Tu, Changlin Zhu

**Affiliations:** College of Field Engineering, Army Engineering University of PLA, Nanjing, China

**Keywords:** Mechanical engineering, Electrical and electronic engineering

## Abstract

This paper proposes a adaptive reaching law-based sliding mode control (SMC) method for maintaining favorable velocity control performance of permanent magnet synchronous motors (PMSMs) under internal and external perturbations. An adaptive switching power reaching law (ASPRL) is designed, which contains adaptive terms and state variables of the sliding mode surface function. This augmented reaching law decreases the chatter of the control system and increases the rate at which the state variables of the system reach the sliding mode surface. Additionally, a Luenberger observer load torque (LOLT) is designed to observe the external load and provide feedback to the velocity controller, reducing the impact of load disturbances and improving the jamming performance of the controller. Simulation experiments confirm that ASPRL reduces buffeting, decreases overshoot, and shortens response time, demonstrating its advantages in PMSM control.

## Introduction

The Permanent Magnet Synchronous Motor (PMSM) is widely used in industrial robots, aerospace, CNC machine tools, and other fields due to its small size, high efficiency, high power density, rapid response speed, and ample torque output. However, PMSM is a complex, multivariable, close-coupled, and nonlinear system that exhibits performance issues with conventional control methods due to parameter mismatch and external interference. Classical control methods such as proportional-integral (PI) controllers are still widely used in industrial production due to their simple structure and convenience. However, PMSM's strong nonlinearity and perturbations make it easy for controllers to deviate from their intended targets due to external disturbances and changes in internal parameters. Linear PI algorithms are inadequate in constraining these perturbations.

In recent years, several nonlinear control methods have been applied to solve the classical linear PI control problem and to control the Permanent Magnet Synchronous Motor (PMSM) in various complex situations. These methods include fuzzy control^1^, adaptive control^2^, predictive control^3^, sliding mode control (SMC)^4–9^, and robust control. These methods have improved the stability of the PMSM system from different aspects. Among these methods, SMC is considered one of the most effective approaches due to its low requirement for model accuracy and insensitivity to perturbations. SMC has been widely used in the field of servomechanism, and researchers have proposed various SMC-based schemes to improve the PMSM system's dynamic performance and suppress the chattering caused by the switching action.

For instance, in Leu et al.^4^, researchers used sliding-mode fuzzy control in the PMSM velocity control system. In Jiang et al.^5^, a predictive control based on SMC is proposed to enhance the velocity robustness and current tracking accuracy of PMSM systems. In Tavoosi^6^, researchers designed a novel radial basis function recursive network (RBFN) to approximate the unknown nonlinear functions in the PMSM dynamics. In Zhang et al.^7^, a memoryless, memory-based sliding integral mode controller is proposed and applied to the control of motor control. In Yong et al.^8^, researchers designed a non-singular SMC at the terminal to apply to uncertain dynamical systems. However, the switching action in sliding mode control causes system chattering, which can deteriorate the dynamic performance of the PMSM system. To solve this problem, researchers have proposed several SMC schemes, such as the high-order sliding mode method^10,11^, fractional sliding mode method^12^, interference observer method^13,14^, reaching law method^15–17^, etc.

Of these, the reaching law method is widely used, and researchers have proposed various reaching law-based schemes to improve the dynamic performance of the PMSM system and suppress chattering. For instance, in Gao and Hung^15^, researchers proposed the reaching law method and analyzed it in three states: steady-state, arrival state, and sliding state. In Junejo et al.^14^, an adaptive terminal glide mode reaching law is designed to reduce approach time and effectively control chatter. In Chen et al.^18^, researchers proposed a scheme based on adaptive fractional-order fuzzy sliding mode control, where sliding mode control is designed using a novel sliding mode fractional integral surface, and adaptive fuzzy control is designed to suppress chatter. In Zhang et al., the traditional power-law reaching law is improved by introducing the exponential term and the state variable of the system, which effectively solves the traditional power-law slow convergence rate problem.

Based on the previous research, this paper aims to improve the dynamic performance of PMSM rate control by proposing an adaptive switching power reaching law (ASPRL) based on the traditional exponential reaching law (TERL). The ASPRL introduces the power term of the system state variable based on adaptation, which improves the system's ability to withstand parameter changes and external interferences, and ensures smooth reaching of the switching surface. Additionally, a Luenberger observer load torque (LOLT) is designed to observe the load and add it to the ASPRL controller as a feedforward compensation, as external load is not measurable. The proposed method is expected to reduce overshoot, chatter, and response time in PMSM control.

The rest of the paper is organized as follows: Section "Adaptive switching power reaching law design" presents the ASPRL and its performance analysis. Section "Design of PMSM sliding mode controller based on ASPRL" discusses the design of the motor control system using the improved reaching law and provides stability proof. Section "Experimental verification" evaluates the ASPRL on the PMSM velocity governing system and compares it with traditional PI control and TERL to verify its performance. Finally, Section "Conclusion" provides a summary of the article.

## Adaptive switching power reaching law design

### Traditional reaching law

SMC is an advanced control mode, which can force the system to move in a low amplitude and high frequency fashion in accordance with the specified trajectory. Because of its small parameter variation and little influence by load disturbance, it is widely used in various complex systems. However, the discontinuous switching nature of SMC will cause system chattering, so the reaching law is often introduced to weaken chattering generated by sliding mode control.

Gao and Hung^15^ first proposed and designed the exponential reaching law, which can be expressed as:

1 $$\frac{ds}{{dt}} = - ks - \varepsilon {\text{sgn}} (s),k > 0,\varepsilon > 0$$

where ${\text{sgn}} (s)$ is symbolic function, $s$ is sliding surface function, $k$ and $\varepsilon$ are symbolic function, $ks$ is exponential approach term, $\varepsilon {\text{sgn}} (s)$ is constant rate reaching term.

In (7), when $s > 0$, can get the following formula:

2 $$\frac{ds}{{dt}} = - \varepsilon - ks$$

And arrival time can be computed by integrating (7) from time 0 to time $t$ where $s(t) = 0$:

3 $$t^{*} = \frac{1}{k}\left\{ {\ln \left[ {s(0) + \frac{\varepsilon }{k}} \right] - \ln \frac{\varepsilon }{k}} \right\}$$

Since the pure exponential reaching law cannot guarantee that the moving point can reach the sliding surface in a bounded amount of time, the constant velocity approach term is added, so that when $s$ is close to 0, the arrival speed $\varepsilon$ is rather than 0. Even though the arrival problem is solved by adding the constant-velocity reaching term, where $k$ determines the exponential law of attaining velocity at the sliding surface, which makes it a contradiction to improve arrival speed and reduce chattering. It can be seen from (9) that to increase the value of arrival speed, the value of $k$ should be increased first. However, too high $k$ value will produce a large velocity to reach the sliding surface, which will cause chattering. It is thus important to balance the arrival rate and suppress chatter, which also provides design ideas for the following reaching law.

### The proposed ASPRL

Based on the above analysis, an adaptive switching power reaching law is proposed:

4 $$\left\{ {\begin{array}{*{20}l} {\dot{s} = \hat{u} - \lambda_{1} \left| x \right|^{\alpha } {\text{sgn}} (s)} \hfill \\ {\alpha = \int {\lambda_{1} \left| s \right| - \int {\lambda_{2} s} } } \hfill \\ \end{array} } \right.$$

where $\hat{u}$ is the nominal reaching law, which can be determined by setting the derivative of the sliding surface equal to 0. The latter is the switching term, where $x$ is the state variable of the system, and $\alpha$ is the adaptive switching power term, can be expressed by the integration of the absolute values of the sliding surface and the sliding surface. Where $\lambda_{1}$ and $\lambda_{2}$ are positive numbers, and $\lambda_{1} > \lambda_{2}$, so $\alpha$ is also positive. In particular, the proposed adaptive sliding mode control law can prevent the integral system from becoming infinite, which is rather different from the conventional sliding mode control law. The sliding mode controller designed in (4) predicts that the sliding mode control law causes the sliding mode surface $s = 0$ to converge in finite time, and the tracking error will asymptotically converge to zero.

The following systems are selected to perform a performance comparison between TERL and ASPRL:

5 $$\ddot{\theta }(t) = - f(\theta ,t) + bu(t)$$

In (11), $f(\theta ,t) = 25\dot{\theta }$ is the actual speed command signal function, $\theta (t)$ is the actual angle command signal, and $b = 133$, $u(t)$ are output control signals.

The $e(t)$ is tracking error, and $e(t)$ the and $\dot{e}(t)$ are defined as:

6 $$\left\{ {\begin{array}{*{20}l} {e(t) = \theta_{d} - \theta (t)} \hfill \\ {\dot{e}(t) = \dot{\theta }_{d} (t) - \dot{\theta }(t)} \hfill \\ \end{array} } \right.$$

where $\theta_{d} (t)$ is the ideal angle signal.

The design sliding mode function is:

7 $$s(t) = ce(t) + \dot{e}(t)$$

where $c$ is sliding surface coefficient, and $c > 0$.

Combining the above equation, we can get:

8 $$\begin{gathered} \dot{s}(t) = c\dot{e}(t) + \ddot{e}(t) = c(\dot{\theta }_{d} (t) - \dot{\theta }(t)) + (\ddot{\theta }_{d} (t) - \ddot{\theta }(t)) \hfill \\ = c(\dot{\theta }_{d} (t) - \dot{\theta }(t)) + (\ddot{\theta }_{d} (t) + f(\theta ,t) - bu(t)) \hfill \\ \end{gathered}$$

It can be seen from Eqs. (10) and (14) that sliding mode control based on the adaptive switching power reaching law can be achieved in the following way:

9 $$\left\{ {\begin{array}{*{20}l} {u(t) = \frac{1}{b}[\lambda_{1} \left| x \right|^{\alpha } {\text{sgn}} (s) - \hat{u} + c(\dot{\theta }_{d} (t) - \dot{\theta }(t) + \ddot{\theta }_{d} (t) + f(\theta ,t)]} \hfill \\ {\alpha = \int {\lambda_{1} \left| s \right| - \int {\lambda_{2} s} } } \hfill \\ \end{array} } \right.$$

The simulation parameters of TERL and ASPRL are set as follows using the S-function of MATLAB:$c = 20$, $\varepsilon = 5$, $k = 5$, $\lambda_{1} = 10$, $\lambda_{2} = 5$. The initial state of the controlled object $x(0)$ is $[x_{1} ,x_{2} ] = [ - 0.15, - 0.15]$, And the ideal position signal of the system is $\theta_{d} = \sin (t)$.

**Figure 1:**
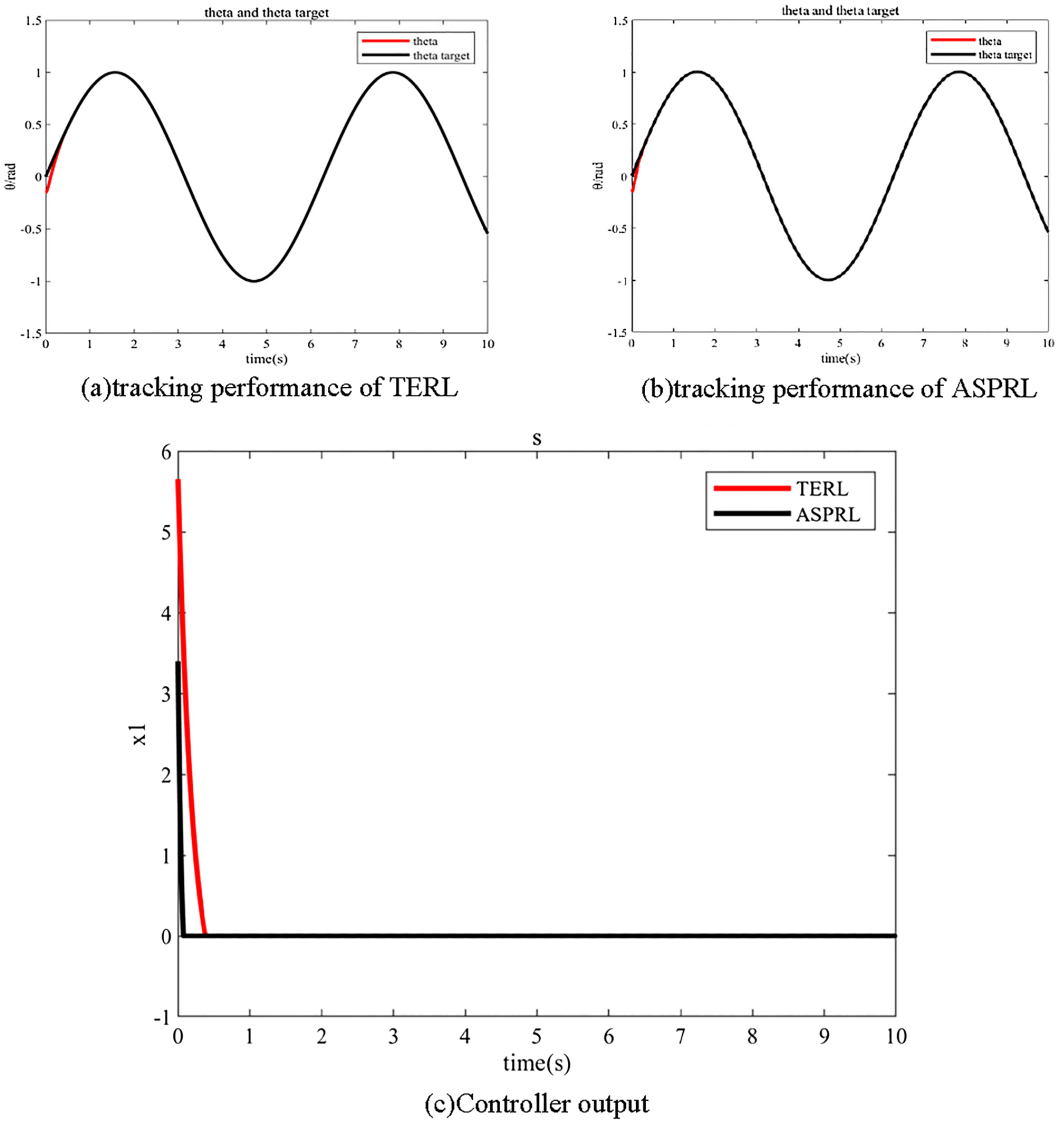
Performance comparison.

The performance comparison between TERL and ASPRL is illustrated in Fig. 1. As seen from Fig. 1a and b, ASPRL outperforms TERL in terms of tracking the reference signal and reducing position error. Moreover, Fig. 1c evidently demonstrate that ASPRL exhibits faster state variable convergence and smoother steady-state process in comparison to TERL. Thus, the proposed ASPRL in this paper is shown to accelerate the approaching velocity and suppress the system chatter, thereby offering significant advantages.

### Discrete forms of ASPRL

In theory, SMC is intended for continuous systems. However, in actual industrial control processes, computer real-time control is used, which transforms the controlled object into a discrete system. In discrete systems, SMC exhibits different stability and characteristics compared to continuous systems. This section compares the performance of ASPRL and TERL in discrete form when the sliding surface is close to 0.

The discrete sliding surface for a given discrete system is designed as follows:

10 $$s(k) = Cx(k)$$

where $$C = [c_{1} ,c_{2} ,...c_{m} ]$$, and $$c_{m} = 0$$.

**Figure 2:**
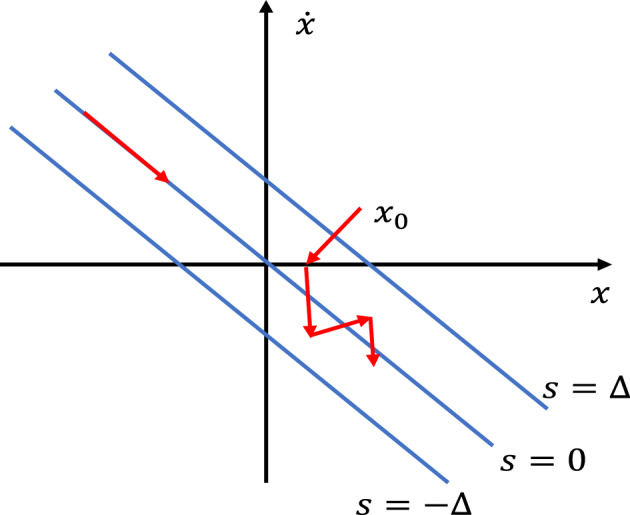
Quasi-sliding mode dynamics.

As sliding mode control cannot generate an ideal sliding mode under discrete form, it can only generate quasi-sliding mode dynamics. Quasi-sliding mode dynamics are defined as follows:

11 $$S^{\Delta } = \{ x \in R^{n} \left| { - \Delta \le s(x) = Cx(k) \le + \Delta } \right.\}$$

where $2\Delta$ represents the bandwidth of the switching band, as shown in the Fig. 2:

If the sliding surface $s$ approaches zero, the ASPRL proposed in (4) can be simplified as $\dot{s} \approx - \varepsilon \left| x \right|^{\alpha } {\text{sgn}} (s)$. The discrete form can be expressed as:

12 $$s_{1} (n + 1) - s_{1} (n) = - T\varepsilon_{1} \left| x \right|^{\alpha } {\text{sgn}} (s_{1} (n))$$

where $T$ is the sampling time.

Assuming the system reaches the slip surface in finite time, that is, the system will reach the slip surface in two trajectories: $s_{1} (0) = 0^{ + }$ and $s_{1} (0) = 0^{ - }$, when $s_{1} (0) = 0^{ + }$, the next periodic equation is:

13 $$s_{1} (n + 1) = - \varepsilon_{1} T\left| x \right|^{\alpha }$$

Similarly, when $s_{1} (0) = 0^{ - }$, the next periodic equation is:NSM

14 $$s_{1} (n + 1) = \varepsilon_{1} T\left| x \right|^{\alpha }$$

According to (13) and (14), the width $\xi_{1}$ of ASPRL discrete sliding mode belt is:

15 $$\xi_{1} = 2\varepsilon_{1} T\left| x \right|^{\alpha }$$

Similarly, the discrete form of TERL is as follows:

16 $$s_{2} (n + 1) - s_{2} (n) = - T\varepsilon_{2} {\text{sgn}} (s_{2} (n))$$

The width $\xi_{2}$ of the discrete sliding mode belt is:

17 $$\xi_{2} = 2\varepsilon_{2} T$$

**Figure 3:**
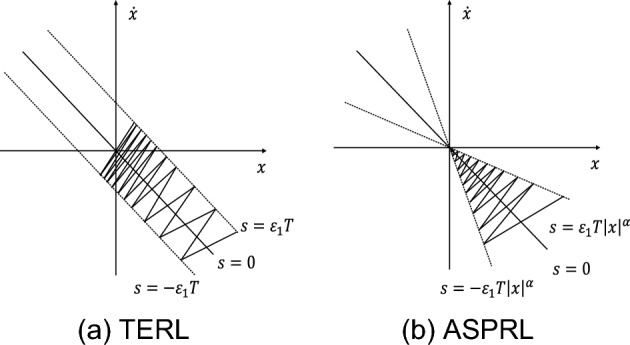
Comparison of state trajectories.

From the analysis shown in Fig. 3, it can be observed that the improved reaching law incorporates the power term of the system's state variable. This modification results in a faster convergence rate and stable convergence to the origin, as evidenced by the switching bandwidth plotted in Fig. 3b. Furthermore, in designing the PMSM speed controller, the speed error is utilized as the system state variable. This approach can potentially reduce chatter and eliminate velocity tracking errors.

## Design of PMSM sliding mode controller based on ASPRL

### Mathematical model of PMSM

PMSM is a strongly coupled multivariable system, and hence, the mathematical model is typically established in the d–q axis of the two-phase rotating reference frame^19^. The establishment of the mathematical model is usually based on the following assumptions:

1. The motor core saturation is neglected;
2. Eddy current and hysteresis losses in the motor are ignored;
3. The current in the motor is a three-phase sinusoidal current with symmetrical components.

Based on the above assumptions, the mathematical model for a three-phase PMSM under the d–q coordinate system has been developed^21^.

Its voltage equation is:

18 $$\left\{ {\begin{array}{*{20}c} {u_{d} = R_{s} i_{d} + \frac{{d\varphi_{d} }}{dt} - \omega_{e} \varphi_{q} } \\ {u_{q} = R_{s} i_{q} + \frac{{d\varphi_{q} }}{dt} + \omega_{e} \varphi_{d} } \\ \end{array} } \right.$$

Flux linkage equation is:

19 $$\left\{ \begin{gathered} \varphi_{d} = L_{d} i_{d} + \varphi_{f} \hfill \\ \varphi_{q} = L_{q} i_{q} \hfill \\ \end{gathered} \right.$$

For surface PMSM $L_{d} = L_{q} = L$, so the torque equation is:

20 $$T_{e} = \frac{3}{2}p\left[ {\varphi_{f} i_{q} + \left( {L_{d} - L_{q} } \right)i_{d} i_{q} } \right]$$

Mechanical equation of motion:

21 $$J\frac{{d\omega_{m} }}{dt} = T_{e} - T_{L} - \eta \omega_{m}$$

According to the above equation, the state space equation is obtained as:

22 $$\left\{ \begin{gathered} \dot{i}_{q} = \frac{1}{{L_{q} }}\left( {u_{q} - R_{s} i_{q} + L_{d} i_{d} \omega_{e} - \psi_{f} \omega_{e} } \right) \hfill \\ \dot{i}_{d} = \frac{1}{{L_{d} }}\left( {u_{d} - R_{s} i_{d} + L_{d} i_{d} \omega_{e} } \right) \hfill \\ \dot{\omega }_{m} = \frac{1}{J}\left( {1.5p\left( {\psi_{f} i_{q} + \left( {L_{d} - L_{q} } \right)i_{d} i_{q} } \right) - T_{L} - \eta \omega_{m} } \right) \hfill \\ \end{gathered} \right.$$

For the purposes of this paper's PMSM vector control scheme, it is assumed $i_{d} = 0$ that the state space Eq. (22) can be rewritten as:

23 $$\left\{ \begin{gathered} \dot{i}_{q} = \frac{1}{{L_{q} }}\left( {u_{q} - R_{s} i_{q} - \psi_{f} \omega_{e} } \right) \hfill \\ \dot{\omega }_{m} = \frac{1}{J}\left( {1.5p\psi_{f} i_{q} - T_{L} - \eta \omega_{m} } \right) \hfill \\ \end{gathered} \right.$$

where $u_{d}$, $u_{q}$ is the voltage of the d–q axis; $i_{d}$, $i_{q}$ is the current of the d–q axis; $L_{d}$, $L_{q}$ is the inductance of the d–q axis; $R_{s}$ is the phase resistance; $P_{n}$ is the number of motor pole pairs; $\varphi_{f}$ It is a permanent magnet synchronous motor permanent magnet flux, $\varphi_{d}$, $\varphi_{q}$ is the component of the permanent magnet flux linkage of the PMSM on the d-q axis; $\omega_{e}$ is the electrical angular velocity; $T_{e}$ is the electromagnetic torque; $\omega_{m}$ is the mechanical angular velocity; $T_{L}$ is the load torque;$J$ is the moment of inertia.

### Design of PMSM speed controller based on ASPRL

Under the condition of $i_{d} = 0$, take the system state variable as Xu^22^:

24 $$\left\{ {\begin{array}{*{20}c} {x_{1} = \omega_{m}^{*} - \omega_{m} } \\ {x_{2} = \dot{x}_{1} = - \dot{\omega }_{m} } \\ \end{array} } \right.$$

where $\omega_{m}^{*}$ is the given speed and $\omega_{m}$ is the actual speed.

Substitute (23) into (24) to get the expression:

25 $$\left\{ {\begin{array}{*{20}l} {\dot{x}_{1} = - \dot{\omega }_{m} = \frac{1}{J}\left( {T_{L} + \eta \omega_{m} - \frac{{3p\psi_{f} }}{2}i_{q} } \right)} \hfill \\ {\dot{x}_{2} = - \ddot{\omega }_{m} = - \frac{{3p\psi_{f} }}{2}\dot{i}_{q} } \hfill \\ \end{array} } \right.$$

If $D = 3p\psi_{f} /2J$, $u = i_{q}$, then the state space expression:

26 $$\left[ {\begin{array}{*{20}c} {\dot{x}_{1} } \\ {\dot{x}_{2} } \\ \end{array} } \right] = \left[ {\begin{array}{*{20}c} 0 & 1 \\ 0 & 0 \\ \end{array} } \right]\left[ {\begin{array}{*{20}c} {x_{1} } \\ {x_{2} } \\ \end{array} } \right] + \left[ {\begin{array}{*{20}c} 0 \\ { - D} \\ \end{array} } \right]u$$

Define sliding surface functions $s$:

27 $$s = cx_{1} + x_{2}$$

where $c$ is the undetermined coefficient.

Then the derivative of the $s$ is:

28 $$\dot{s} = cx_{2} - Du$$

Let $\dot{s} = 0$ to find:

29 $$\hat{u} = \frac{c}{JD}\left( {T_{L} + \eta \omega_{m} - D} \right)$$

Substituting Eq. (29) into (4), we can get:

30 $$u = \hat{u} - \lambda_{1} \left| x \right|^{\alpha } {\text{sgn}} (s)$$

where $\alpha$ is the adaptive switching power term. The parameter selection is:

31 $$\alpha = \int {\lambda_{1} \left| s \right| - \int {\lambda_{2} s} }$$

Substituting ASPRL (4) into (30) gives:

32 $$\hat{u} - \lambda_{1} \left| x \right|^{\alpha } {\text{sgn}} (s) = cx_{2} - Du$$

To sum up, the output signal $i_{q}$ of the speed loop controller can be obtained:

33 $$i_{q} = \frac{1}{D}\int {cx_{2} - \hat{u} + \lambda_{1} \left| x \right|}^{\alpha } {\text{sgn}} (s)d\tau$$

### Stability analysis

Define Lyapunov function as:

34 $$V = \frac{1}{2}s^{2}$$

Differentiate it and substitute it into the reaching law to get:

35 $$\dot{V} = s\dot{s} = s[ - \lambda_{1} \left| x \right|^{\alpha } {\text{sgn}} (s)] = - \lambda_{1} \left| x \right|^{\alpha } s \cdot {\text{sgn}} (s)$$

where when the parameter selection meets $\lambda_{1} > \lambda_{2} > 0$, then $\alpha > 0$, $s \cdot {\text{sgn}} (s) > 0$, and $\dot{V} \le 0$, From Lyapunov stability theory, it can be seen that the designed sliding mode controller is stable, which guarantees that the mobile points of the system reach the sliding mode surface within a finite amount of time.

### Design of Luenberger observer load torque

In the design of a real motor controller, load perturbation must be taken into consideration. Since velocity can be measured, but load torque cannot, a load torque observer needs to be designed to provide feedback on the observed value of the load torque to the motor controller. This is done in order to minimize the impact of external loads on the motor system.

Torque $T_{e}$ as system input, and $\omega_{m}$ as system output, speed $\omega_{m}$ and external load $T_{L}$ are selected as state variables. The state space expression is:

36 $$\begin{aligned} \left[ {\begin{array}{*{20}c} {\dot{\omega }_{m} } \\ {\dot{T}_{L} } \\ \end{array} } \right] & = \left[ {\begin{array}{*{20}c} { - \frac{\eta }{J}} & { - \frac{1}{J}} \\ 0 & 0 \\ \end{array} } \right]\left[ {\begin{array}{*{20}c} {\omega_{m} } \\ {T_{L} } \\ \end{array} } \right] + \left[ {\begin{array}{*{20}c} \frac{1}{J} \\ 0 \\ \end{array} } \right]T_{e} , \\ y & = \left[ {\begin{array}{*{20}c} 1 & 0 \\ \end{array} } \right]\left[ {\begin{array}{*{20}c} {\omega_{m} } \\ {T_{L} } \\ \end{array} } \right] \\ \end{aligned}$$

The LTLO equation can be described as:

37 $$\left\{ {\begin{array}{*{20}l} {\dot{\hat{x}} = A\hat{x} + Bu + L(y - \hat{y})} \hfill \\ {\hat{y} = C\hat{x}} \hfill \\ \end{array} } \right.$$

where $L$ is the coefficient matrix, and

38 $$\begin{gathered} L = \left[ {\begin{array}{*{20}c} {L_{1} } \\ {L_{2} } \\ \end{array} } \right],A = \left[ {\begin{array}{*{20}c} {\frac{ - \eta }{J} - L_{1} } & {\frac{ - 1}{J}} \\ { - L_{2} } & 0 \\ \end{array} } \right] \hfill \\ B = \left[ {\begin{array}{*{20}c} \frac{1}{J} \\ 0 \\ \end{array} } \right],C = \left[ {\begin{array}{*{20}c} 1 & 0 \\ \end{array} } \right] \hfill \\ \end{gathered}$$

Therefore, Eq. (37) can be described as:

39 $$\dot{\hat{x}} = (A - LC)\hat{x} + Bu + Ly$$

Substituting Eq. (38) into it, we can get:

40 $$\left[ {\begin{array}{*{20}c} {\dot{\hat{\omega }}_{m} } \\ {\dot{\hat{T}}_{L} } \\ \end{array} } \right] = \left[ {\begin{array}{*{20}c} { - \frac{\eta }{J} - L_{1} } & { - \frac{1}{J}} \\ { - L_{2} } & 0 \\ \end{array} } \right]\left[ {\begin{array}{*{20}c} {\hat{\omega }_{m} } \\ {\hat{T}_{L} } \\ \end{array} } \right] + \left[ {\begin{array}{*{20}c} \frac{1}{J} \\ 0 \\ \end{array} } \right]T_{e} + \left[ {\begin{array}{*{20}c} {L_{1} } \\ {L_{2} } \\ \end{array} } \right]\omega_{m}$$

Equation (40) is further simplified as:

41 $$\left\{ {\begin{array}{*{20}l} {\dot{\hat{\omega }}_{m} = \frac{1}{J}(T_{e} - \hat{T}_{L} - \eta \hat{\omega }_{m} + JL_{1} (\omega_{m} - \hat{\omega }_{m} ))} \hfill \\ {\dot{\hat{T}}_{L} = L_{2} (\omega_{m} - \hat{\omega }_{m} )} \hfill \\ \end{array} } \right.$$

To make the observed value approach the true value, the eigenvalue of matrix $A - LC$ must be less than 0. To find the eigenvalues of the matrix $A - LC$:

42 $$\left| {\lambda I - \left[ {\begin{array}{*{20}c} { - \frac{\eta }{J} - L_{1} } & { - \frac{1}{J}} \\ { - L_{2} } & 0 \\ \end{array} } \right]} \right| = 0$$

Modify Eq. (42) to the following form:

43 $$\lambda^{2} + (\frac{\eta }{J} + L_{1} )\lambda - \frac{{L_{2} }}{J} = 0$$

Define the characteristic value $\lambda$ is $a_{1} ,a_{2}$, and get:

44 $$\lambda^{2} - (a_{1} + a_{2} )\lambda + a_{1} a_{2} = 0$$

where $(a_{1} < 0,a_{2} < 0)$, by comparing (43) and (44), we can get:

45 $$\left\{ {\begin{array}{*{20}l} {L_{1} = - (a_{1} + a_{2} + \frac{\eta }{J})} \hfill \\ {L_{2} = - a_{1} a_{2} J} \hfill \\ \end{array} } \right.$$

By changing the value of $a_{1} ,a_{2}$, adjusted the speed of the charging observer as it approaches the true value. The observed load torque $\hat{T}_{L}$ is the feedback to the velocity controller, and the expression for the controller is:

46 $$i_{q} = \frac{2J}{{3P\varphi_{f} }}\int {cx_{2} - } \frac{2c}{{3P\varphi_{f} }}(\hat{T}_{L} + \eta \omega_{m} ) + \frac{c}{J} + \lambda_{1} \left| x \right|^{\alpha } {\text{sgn}} (s)d\tau$$

## Experimental verification

**Figure 4:**
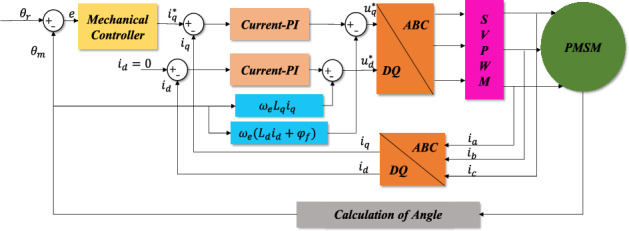
PMSM vector control structure diagram.

To confirm the validity of the proposed approach, this paper utilized the control scheme displayed in Fig. 4 for a simulation and analysis through a Matlab/Simulink model. Moreover, the physical system was constructed on TI company's TMS320F28035 development kit as the control chip, and was compared to PI control and exponential approach law sliding mode control for experimental validation. The motor parameters are presented in Table 1.

**Table 1:** Permanent magnet synchronous motor parameters.

Parameter	Value
Inductance $L_{d} /mH$	5.25
Inductance $L_{q} /mH$	12
Inertia J/kg m^2^	0.003
Stator resistance $R/\Omega$	0.985
Motor pole pairs $P$	4
Permanent magnet flux linkage $\varphi_{f} /wb$	0.1827

The parameters of the speed PI controller are: $K_{p} = 0.14$, $K_{i} = 7$. The parameters of the TERL are: $c = 70$, $q = 800$, $\varepsilon = 200$. The parameters of the ASPRL + LTLO controller are: $a_{1} = - 800$, $a_{2} = - 510$, $\lambda_{1} = 100$, $\lambda_{2} = 10$, $c = 0.5$, $q = 800$, $\varepsilon = 100$.

The motor parameters used in the experiment are shown in Table 1.

**Figure 5:**
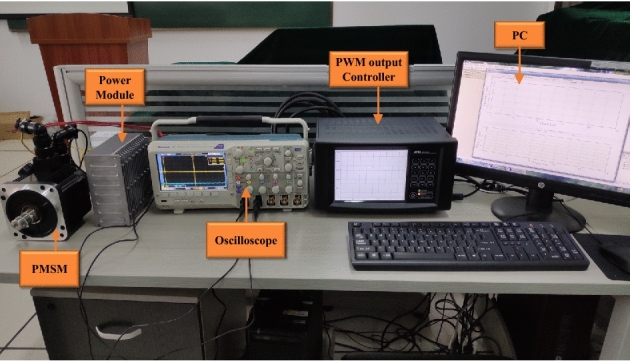
PMSM control platform.

The PMSM controller platform is illustrated in Fig. 5. The control program is written and burned into the control unit from a PC. Based on the program, the control unit generates the SVPWM control signal and sends it to the power module for driving the PMSM. In addition, the control unit may receive feedback signals and send them back to the computer. The power module produces a three-phase SVPWM wave based on the received SVPWM control signal. An oscilloscope is used to assist in inspection.

**Figure 6:**
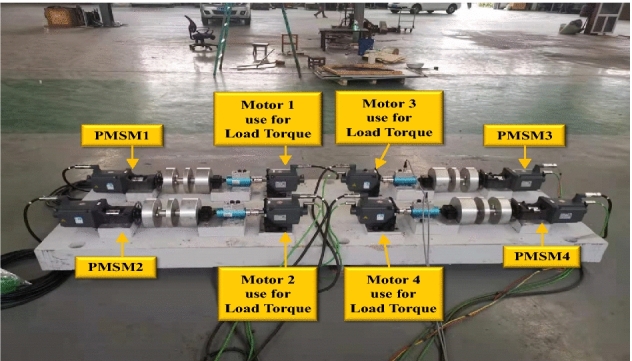
PMSM test platform.

The PMSM testing and control platform is shown in Fig. 6. The system can operate according to the PMSM control platform described earlier. The system comprises 4 PMSMs as sensing engines, while the other 4 PMSMs are used to output the specified constant torque as the torque due to loading. Other sensors in the system include a torque sensor, an ammeter, a voltmeter, among others. In this experiment, PMSM1 is controlled by a PI controller, PMSM2 is controlled by TERL, and PMSM3 is controlled by the ASPRL controller.

**Figure 7:**
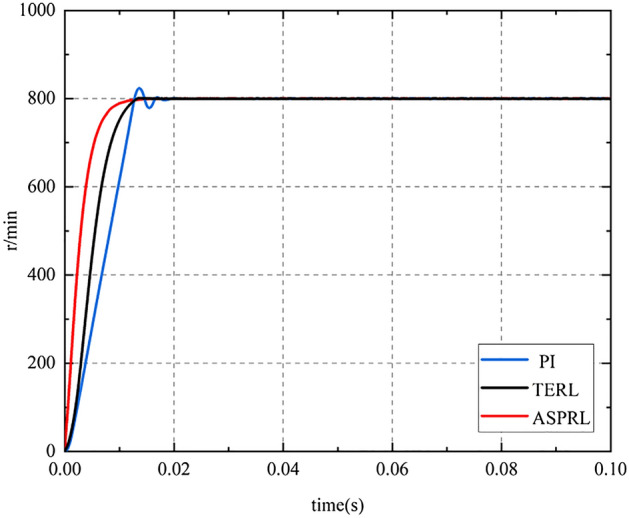
The speed response under three control methods with no load.

To validate the dynamic performance of the controller, the first experiment extensively compared the startup process of the PMSM under different control schemes when the given reference speed was set to 800r/min and the initial load torque was 0 Nm. Figure 7 illustrates the speed response during the startup process based on the PI, TERL, and ASPRL controllers.

From the graph, it can be concluded that both the TERL and ASPRL control methods exhibit rapid motor response, reaching steady-state in 0.13 s and 0.12 s, respectively, and maintaining stability thereafter. The motor response under the PI control method is relatively slow, requiring a steady-state time of 0.18 s after experiencing oscillations.

In order to compare the effect of control performance more clearly and intuitively, integral absolute error (IAE) is used to calculate the speed response during startup,The IAE can be expressed as:

$$\omega = \frac{{\sqrt{\frac{1}{N}} \sum\limits_{i = 1}^{N} {(\omega_{m} - \omega_{a} )^{2} } }}{{\omega_{a} }}$$

where $\omega_{m}$ is instantaneous response speed; $\omega_{a}$ is reference speed.

As shown in Table 2, compare the dynamic stability performance of the control algorithm.

**Table 2:** Startup performance comparison.

	IAE Index	Settling time (s)
PI	4132	0.18
TERL	3093	0.13
ASPRL	1591	0.12

**Figure 8:**
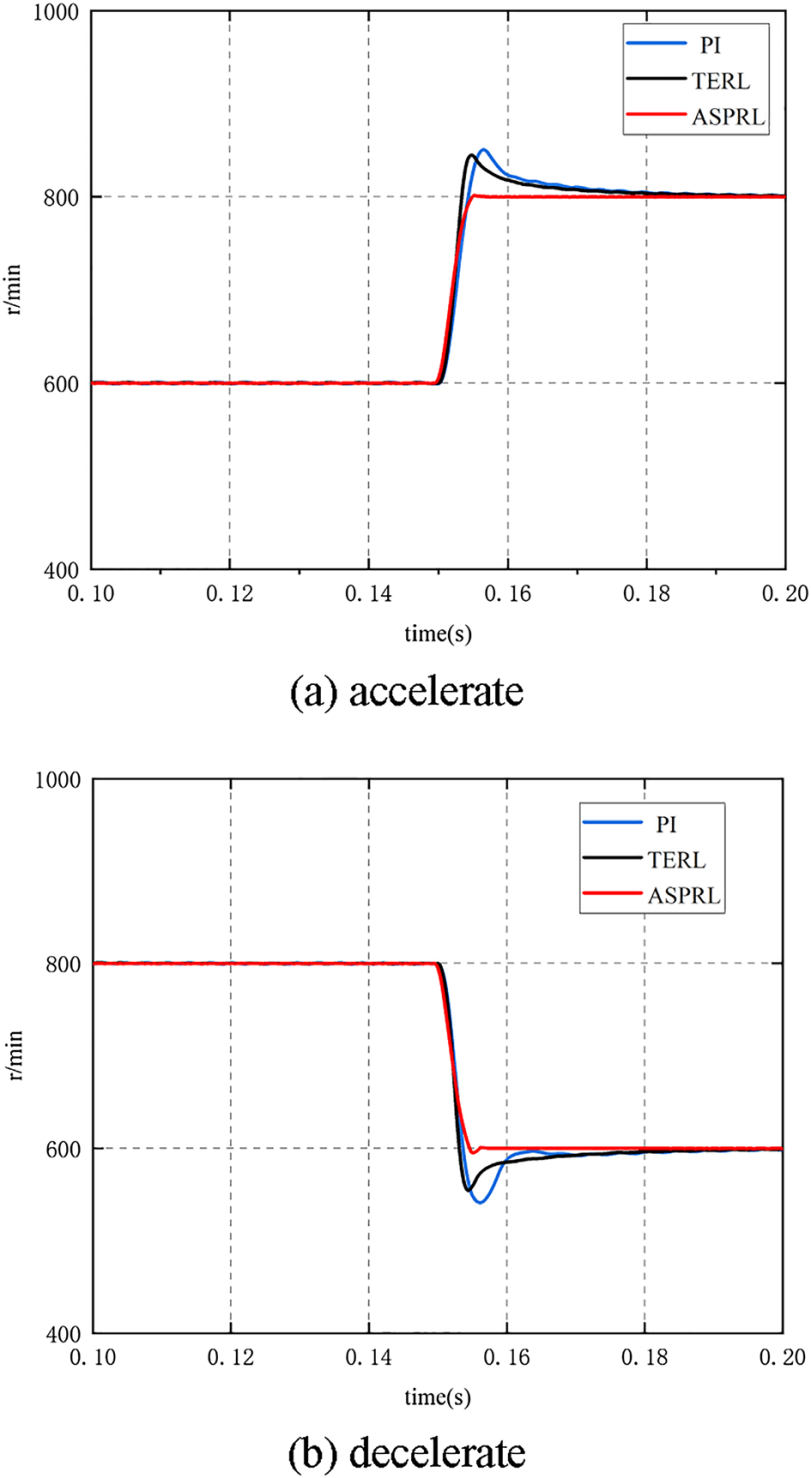
The variable acceleration responses under three control methods.

To verify the controller's control performance under speed step changes, the speed variations of the PMSM under the PI, TERL, and ASPRL controllers were compared. The speed variations were from 600 to 800 r/min and from 800 to 600 r/min, as shown in Fig. 8a and b. From the results, it can be concluded that during the speed variation process, the PI controller had overshoots of 6% and 9.8% respectively, the TERL controller had overshoots of 5% and 9% respectively, while the ASPRL controller had almost no overshoot. Therefore, the ASPRL controller has a smaller steady-state error range and lower speed fluctuations.

During the sudden change of speed, the IAE indexes of PI, terl and asprl are shown in Table 3 respectively.

**Table 3:** Performance comparison during speed change.

	600–800		800–600	
	IAE Index	Settling time (s)	IAE Index	Settling time (s)
PI	154	0.041	194	0.045
TERL	129	0.037	165	0.039
ASPRL	96	0.013	130	0.016

**Figure 9:**
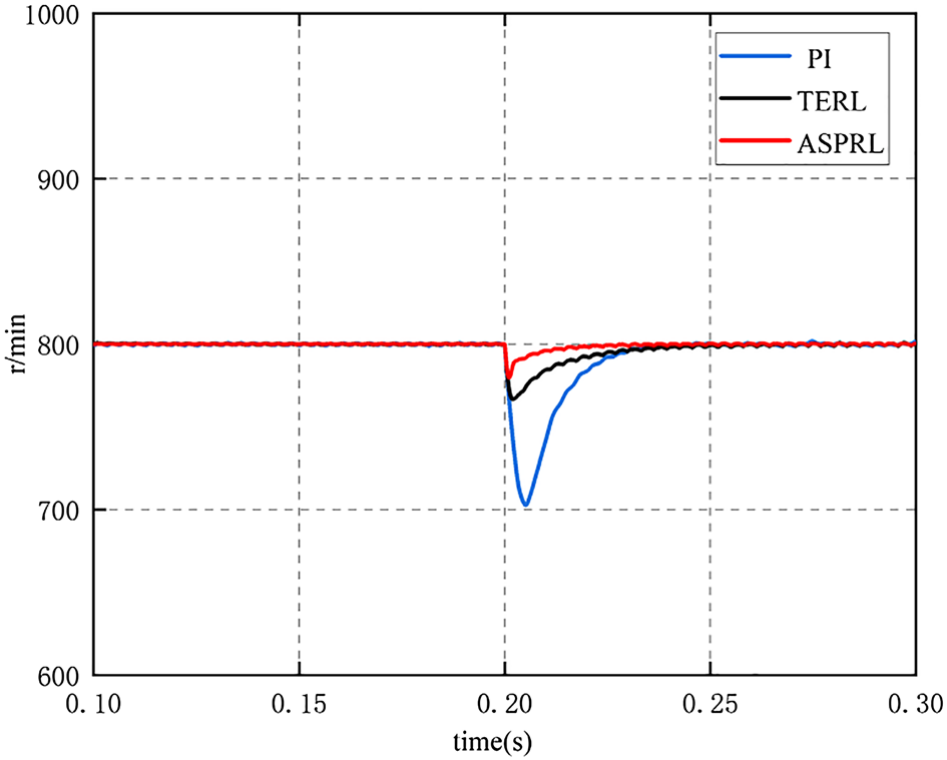
The speed response under three control methods with the load of 10 Nm.

**Figure 10:**
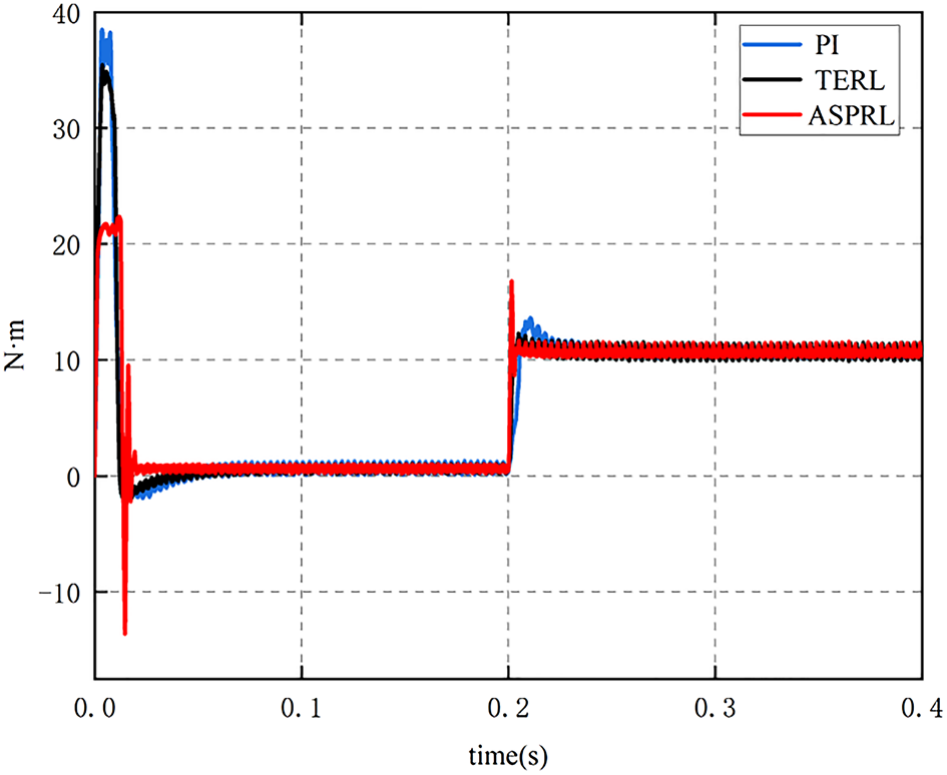
The torque response under three control methods with the load of 10 Nm.

**Figure 11:**
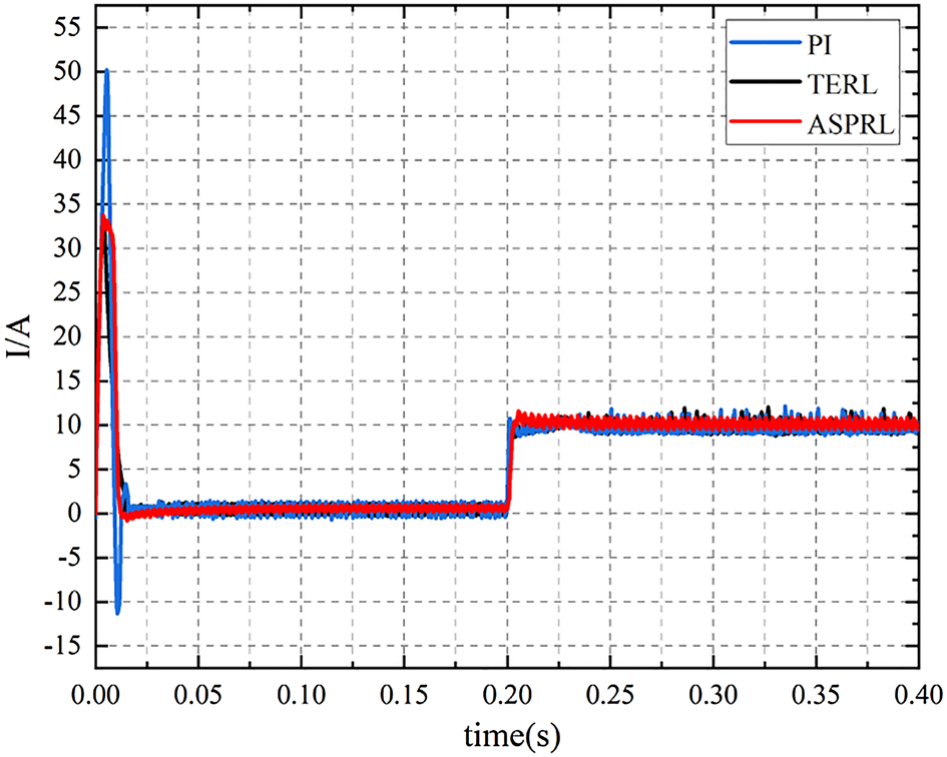
$$i_{q}$$ current curves.

To evaluate the controller’s ability to resist load disturbances when the PMSM is in operation, a reference speed of 800 r/min was given. At 0.2 s into the PMSM operation, a load torque of 10 Nm was applied, and the speed and torque variations of the PMSM were compared under different control schemes. As shown in Fig. 9, when the external load is 10 Nm, the speed reaches steady-state in 0.262 s for the PI controller, 0.234 s for TERL, and 0.216 s for ASPRL. Furthermore, the speed drops by 98 rpm, 33 rpm, and 8 rpm, respectively, for the different control schemes. Figures 10 and 11 illustrate the torque and current variations under the external load conditions. It can be concluded that the ASPRL controller reaches the stable state fastest when subjected to a load, and it exhibits excellent disturbance rejection capabilities.

The IAE indexes of PI, terl and asprl are shown in Table 4 respectively.

**Table 4:** Performance comparison during speed change.

	IAE Index	Speed drop (rpm)
PI	121	98
TERL	25	33
ASPRL	11	8

## Conclusion

This paper introduces a control approach called the adaptive switching power reaching law (ASPRL), which is designed to address the limitations of the traditional sliding mode control law (TERL), such as slow convergence and excessive chattering. ASPRL is shown to improve the arrival rate of the PMSM during startup and load transients, as well as effectively suppress chattering. Additionally, to address the issue of unmeasurable external loads, a Luenberger observer load torque (LOLT) is proposed. The combination of ASPRL and LOLT further enhances anti-jamming capability and eliminates chattering. The stability of the closed loop control system is confirmed using Lyapunov functions. The paper provides a detailed comparison of the anti load perturbation capability of the three proposed methods: ASPRL + LOLT, traditional PI, and TERL. Experiments and simulations are conducted to verify the effectiveness of the proposed ASPRL + LOLT method. The experimental results demonstrate that this method provides higher quality velocity control, better anti-interference, and faster chattering suppression capability.

## Data Availability

All data generated or analysed during this study are included in this published article.
